# The majority of patients with newly diagnosed juvenile idiopathic arthritis achieve a health-related quality of life that is similar to that of healthy peers: results of the German multicenter inception cohort (ICON)

**DOI:** 10.1186/s13075-018-1588-x

**Published:** 2018-05-30

**Authors:** Miriam Listing, Kirsten Mönkemöller, Ina Liedmann, Martina Niewerth, Claudia Sengler, Joachim Listing, Dirk Foell, Arnd Heiligenhaus, Ariane Klein, Gerd Horneff, Gerd Ganser, Johannes-Peter Haas, Jens Klotsche, Kirsten Minden

**Affiliations:** 1 Deutsches Rheuma-Forschungszentrum Berlin, Epidemiology Unit, Charitéplatz 1, 10117 Berlin, Germany; 2 Kinderkrankenhaus Amsterdamer Straße, Kliniken der Stadt Köln gGmbH, Köln, Germany; 3 Department of Pediatric Rheumatology and Immunology, University of Münster, Münster, Germany; 4 Department of Ophthalmology at St. Franziskus Hospital Münster, University of Duisburg-Essen, Duisburg, Germany; 5 Asklepios Klinik Sankt Augustin, Sankt Augustin, Germany; 6 St. Josef-Stift Sendenhorst, Sendenhorst, Germany; 7 Deutsches Zentrum für Kinder- und Jugendrheumatologie, Garmisch-Partenkirchen, Germany; 8 Charité – Universitätsmedizin Berlin, Department of Rheumatology and Clinical Immunology, Berlin, Germany

**Keywords:** Inception cohort, Juvenile idiopathic arthritis, Quality of life, Outcome

## Abstract

**Background:**

Achieving the best possible health-related quality of life (HRQoL) for a patient is an important treatment goal in juvenile idiopathic arthritis (JIA). We investigated the 36-month trajectories of HRQoL in children with JIA compared with healthy peers and identified the predictors of an unfavorable HRQoL.

**Methods:**

Patients with a recent JIA diagnosis were enrolled in the German inception cohort study ICON. As a peer group, friends of patients of the same age and sex were asked to cooperate. Children were prospectively followed and regularly questioned about their HRQoL using the Pediatric Quality of Life Inventory 4.0 (PedsQL). Disease activity was assessed by the clinical Juvenile Arthritis Disease Activity Score (cJADAS-10), and the burden of the child’s chronic illness on their family was assessed by the Family Burden Questionnaire (FaBel). Linear mixed models were used to compare the HRQoL of the patients and their peers. Associations between the health status of a patient at enrollment and an unfavorable HRQoL (PedsQL total < 79.3) at their 3-year follow-up (FU) were analyzed by logistic regression.

**Results:**

Data from 953 patients (median symptom duration 6 months, mean age 7.9 years) and 491 healthy peers (aged 8.4 years) were analyzed. During 3 years of FU, the disease activity and HRQoL of the patients improved significantly (cJADAS-10 from 9.8 (6.2) to 2.7 (3.6) and PedsQL total score from 71.7 (18.2) to 87.3 (13.9)). While the HRQoL of the patients varied among the several JIA categories at the time of enrollment, no significant differences were found at the 3-year FU. After 36 months, the HRQoL of the patients had largely converged with that of their healthy peers. JIA patients had a psychosocial health status comparable with their healthy peers, whereas a small significant mean difference remained in physical health (5.8, 95% confidence interval (CI) 4.1–7.6). Up to the 36-month FU, three-quarters of JIA patients attained a favorable HRQoL (PedsQL ≥ 79.3) which was achieved by 90% of the peers. A higher family burden, higher pain level, and lower well-being at enrollment were associated with an unfavorable HRQoL.

**Conclusions:**

Under current therapeutic conditions, an HRQoL corresponding with that of healthy children is a realistic treatment goal in JIA.

## Background

Juvenile idiopathic arthritis (JIA) is the most common chronic inflammatory rheumatic illness in children, with an incidence of 2–20 cases per 100,000 at-risk children [1]. JIA encompasses a clinically heterogeneous group of disorders with unknown causes which begin before the age of 16 years. These diseases cause both temporary and permanent disability as well as an impaired quality of life [2–4]. The treatment of JIA currently aims at achieving an inactive disease state, preventing disability and damage, and ensuring the age-appropriate development of affected children and adolescents. As JIA influences virtually all aspects of the child’s life and those of his or her family, achievement of an optimal health-related quality of life (HRQoL) is an important goal in clinical care. HRQoL is a complex, multidimensional concept that encompasses physical, emotional, social, and behavior-related well-being and functioning. A patient’s individual perception of health is affected by his/her social and cultural background as well as his/her personal value system [5–7]. Several studies [4, 8–15] have shown that JIA patients have a lower HRQoL compared with healthy controls. Most prior studies were cross-sectional in their study design [8, 12–14], and the assessment of HRQoL was often conducted several years after diagnosis [4, 11]. Longitudinal studies of HRQoL in JIA patients are rare [4, 16–18], and some were performed to examine the effects of disease-modifying antirheumatic drugs (DMARDs) [16–18].

A large Canadian inception cohort [19] investigated changes in the HRQoL of patients with JIA. They found that the HRQoL of children with JIA improved gradually over time. Another inception cohort from Scandinavia [20] found reduced physical health in only 20% of JIA patients approximately 8 years after disease onset. Some indicators of a poor quality of life have been identified [9, 21–24] and include the persistence of a high level of disease activity, the presence of disabilities, chronic pain, and low social or emotional resources. However, some questions remain unanswered; for example, it has remained unclear why patients with clinically inactive disease nevertheless report a reduced quality of life [25]. Identifying the factors that predict a poor outcome with continued low HRQoL is a vital step toward providing appropriate interventions.

To the best of our knowledge, there are no published data that evaluate the course of HRQoL in newly diagnosed JIA patients compared with healthy peers. We therefore used data from the German Inception Cohort Of patients with Newly diagnosed JIA (ICON) to investigate the HRQoL of affected children during the first years of pediatric rheumatologic care in order to: 1) describe changes in HRQoL over time; 2) compare the HRQoL of patients with that of healthy peers; and 3) identify the predictors of a suboptimal HRQoL in the course of the disease.

## Methods

### Study design

ICON is an ongoing, prospective, observational cohort study into which patients with a recent onset of JIA and healthy controls were enrolled from May 2010 to December 2012 at 11 of the largest pediatric rheumatology sites in Germany. For the recruitment of the comparator group, patients and/or their parents were asked to recruit some of their friends of equal age and gender from kindergarten or school to participate as healthy peers. Informed consent was obtained from children (≥ 8 years) and their parents. The study protocol was approved by the ethics committee of the Charité – University Medicine Berlin. For more details on the ICON cohort study, see Sengler et al. [26].

### Participants

Patients had to have a diagnosis of JIA (according to the International League of Associations for Rheumatology (ILAR) classification criteria [27]) for less than 12 months. A total of 953 consecutively observed patients with confirmed JIA and 491 healthy peers were included in the ICON study. Data available by 21 June 2017 were used for this analysis. After enrollment, JIA patients were assessed every 3 months in the first year and every 6 months thereafter, while healthy peers were questioned once a year. For this analysis, the 36-month follow-up (FU) was chosen as the study endpoint.

### Measurements

Demographic data (age, sex, parent education, income, country of origin, and actual place of residence) were reported by the parents of the JIA patients and their healthy peers. At each study visit, the pediatric rheumatologist recorded treatment modalities and assessed the patient’s disease state, for example the number of active (range 0–70) joints, and disease activity (physician’s global assessment of disease activity (PGA)) on a 21-point numeric rating scale (NRS; 0–10). Parents of the JIA patients and their healthy peers assessed their child’s overall well-being (parent’s global assessment) and pain on a 21-point NRS (0–10) at each visit. Laboratory parameters such as the erythrocyte sedimentation rate (ESR), C-reactive protein (CRP), antinuclear antibodies (ANA), rheumatoid factor (RF), and human leukocyte antigen-B27 (HLA-B27) were also recorded. JIA categories according to ILAR criteria were reported by physicians at enrollment and 1-year and 3-year FU and were reviewed by two pediatric rheumatologists (KM and CS) [26, 27].

JIA disease activity was evaluated by the clinical Juvenile Arthritis Disease Activity Score (cJADAS-10). The cJADAS-10 (range 0–30) is calculated with the physician’s global assessment, the parent’s global assessment, and the number of actively involved joints (maximum 10). The cJADAS was found to be valid, feasible, and applicable in all JIA categories [28]. The cJADAS-10 thresholds proposed by Consolaro et al. [29, 30] were applied to define disease activity state (inactive disease, and minimal, moderate, or high disease activity). In patients with oligoarticular disease course (≤ 4 active joints), the cut-off points 1, 1.5, and 4 were used. The thresholds 1, 2.5, and 8.5 were used in those with polyarticular disease course (> 4 active joints).

The Childhood Health Assessment Questionnaire (CHAQ) [31] was used to measure the functional ability of the patients. To evaluate the HRQoL in JIA patients and healthy peers, the German version of the Pediatric Quality of Life Inventory generic core scales 4.0 (PedsQL) was used [32, 33]. The PedsQL consists of four subscales (physical functioning, emotional functioning, social functioning, and school functioning) which can be combined into physical health and psychosocial health composite scales, as well as the PedsQL total score. PedsQL scores range from 0 (being the worst) to 100 (being the best possible HRQoL). The parents of all participants completed the PedsQL at each visit.

The Strength and Difficulties Questionnaire (SDQ) [34, 35] was administered to the parents of all children aged 5 years or above at the time of enrollment. The total difficulties score ranges from 0 to 40. Higher scores are an indicator of more severe psychosocial problems, e.g., emotional and behavioral problems: scores ≤ 13 are classified as ‘normal’; scores 14–16 as ‘borderline’; and scores ≥ 17 as ‘conspicuous’.

Parents of JIA patients also completed the German version of the Impact on Family Scale [36], the Family Burden Questionnaire (FaBel) 3 months after enrollment. The FaBel [37] measures the burden of a chronic illness on the family of the patient. The summary scale ranges from 0 to 4, and higher scores indicate a greater family burden.

### Socioeconomic status

An established German multidimensional aggregated index was used to measure the socioeconomic status (SES) of the child. This index was evaluated and adapted from a representative German population sample of 17,641 study participants aged up to 17 years [38]. Since the parental work status was not ascertained in the ICON study, the calculation of this index was modified to be based only on parental education level (including schooling and vocational training) and household net income. According to Lampert et al. [38], the highest education level of the mother or father was used to assign the specific education score (ranging from low (1) to high (7)). The household equivalence net income score was calculated by dividing the net income by the square root of the number of family members (range from low (1) to high (7)) (http://www.oecd.org/els/soc/OECD-Note-EquivalenceScales.pdf). Based on the education level of the parents, missing net income data were imputed separately for parents of patients and parents of peers using the SAS® procedure MI. One imputation was used to calculate the household equivalence net income score since this is not a main outcome parameter. The lower and upper quartiles of the sum of the education and income scores (6.55, 12.1) were used as cut-points to define low, moderate, and high SES.

### Study questions

The PedsQL total score and the PedsQL physical health as well as psychosocial health scales were used to compare the 36-month trajectories of HRQoL of JIA patients with that of healthy peers (aims 1 and 2, respectively). PedsQL scores of the peers were used to define optimal and suboptimal HRQoL states. Predictors of a suboptimal HRQoL status were identified in JIA patients (aim 3). In addition, as secondary outcomes, the disease activity measured by the cJADAS-10 and functional ability assessed by the CHAQ were investigated.

### Statistical analysis

Marginal structural models were applied to achieve an approximation of age and gender distribution and social status characteristics between patients and peers. The following approach was applied. Logistic regression with the binary dependent variable patient or peer and the covariables age, sex, country of origin, parental education, and household equivalence net income was used to calculate weighted samples. For example, peers with a low social status underrepresented in the peer group were given a weight > 1, while the overrepresented group of peers with a high social status received a weight < 1. This weighting resulted in balanced samples which were used for the statistical analysis. Only columns 2 and 3 of Table 1 and Table 2 refer to unweighted samples; all other analyses have been calculated on the basis of weighted samples.

**Table 1:** Demographic data and proxy-reported health-related quality of life outcomes of patients and healthy peers at baseline

	Patients (*n* = 953)	Peer group (*n* = 491)	Weighted patients (*n* = 953)	Weighted peers (*n* = 491)
Female, *n* (%)	640 (67.2%)*	290 (59.1%)	613 (64.3%)	311 (64%)
Age at study enrollment (years), mean (SD)	7.9 (4.8)*	8.4 (4.6)	8.1 (4.9)	8.3 (4.5)
Country of origin Germany, *n* (%)	697 (76.9%)**	413 (84.5%)	726 (79.8%)	390 (80.6%)
Parent’s education score, mean (SD)	4.7 (1.6)**	5.4 (1.5)	5.0 (1.6)	5.0 (1.5)
Equivalence household net income score, mean (SD)	4.0 (1.9)**	4.7 (1.8)	4.2 (1.9)	4.2 (1.9)
SES, mean (SD)	8.7 (3.0)**	10.1 (2.9)	9.2 (3.1)	9.3 (3.0)
Low (≤ 6.55), *n* (%)	288 (30.2%)	78 (15.9%)	241 (25.3%)	120 (24.7%)
Moderate, *n* (%)	486 (51.0%)	238 (48.5%)	485 (50.8%)	239 (49.3%)
High (≥ 12.1), *n* (%)	179 (18.8%)	175 (35.6%)	229 (24.0%)	126 (26.0%)
PedsQL total score, mean (SD)	71.5 (18.4)**	89.9 (7.7)	71.7 (18.2)**	89.6 (7.9)
PedsQL physical health, mean (SD)	66.0 (24.6)**	95.1 (7.1)	66.4 (24.4)**	94.9 (7.2)
PedsQL psychosocial health, mean (SD)	74.8 (17.4)**	87.1 (9.5)	74.9 (17.3)**	86.7 (9.7)
PedsQL emotional functioning, mean (SD)	68.9 (22.1)**	80.4 (13.8)	69.0 (21.9)**	80.1 (14.1)
PedsQL school functioning, mean (SD)	72.9 (20.9)**	88.1 (12.1)	73.1 (20.8)**	87.6 (12.3)
PedsQL social functioning, mean (SD)	82.0 (19.1)**	92.7 (10.0)	82.0 (19.0)**	92.5 (10.0)
SDQ, total scale (range 0–40), mean (SD)	9.4 (5.8)**	5.8 (4.8)	9.2 (5.9)**	6.1 (4.9)
Parent’s global (NRS 0–10), mean (SD)	3.0 (2.3)**	0.7 (1.0)	3.0 (2.3)**	0.7 (1.0)
Pain (NRS 0–10), mean (SD)	3.0 (2.8)**	0.4 (0.8)	2.9 (2.8)**	0.4 (0.9)

*p < 0.05, **p < 0.001

NRS, numeric rating scale; PedsQL, Pediatric Quality of Life Inventory; SD, standard deviation; SDQ, Strength and Difficulties Questionnaire; SES, socioeconomic status

**Table 2:** Characteristics of JIA patients at baseline, and at the 1-year, 2-year, and 3-year follow-up (FU)

	Baseline	1-year FU	2-year FU	3-year-FU
*n*	953	850	805	761
JIA categories				
Oligoarthritis, *n* (%)	441 (46.3%)	–	–	–
Persistent oligoarthritis, *n* (%)		315 (37.1%)	–	253 (33.3%)
Extended oligoarthritis, *n* (%)		74 (8.7%)	–	98 (12.9%)
RF-negative polyarthritis, *n* (%)	250 (26.2%)	226 (26.6%)	–	205 (27.0%)
RF-positive polyarthritis, *n* (%)	16 (1.7%)	15 (1.8%)	–	15 (2.0%)
Enthesitis-related arthritis, *n* (%)	100 (10.5%)	92 (10.8%)	–	75 (9.9%)
Psoriatic arthritis, *n* (%)	40 (4.2%)	41 (4.8%)	–	35 (4.6%)
Systemic-onset JIA, *n* (%)	35 (3.7%)	29 (3.4%)	–	28 (3.7%)
Undifferentiated arthritis, *n* (%)	71 (7.5%)	58 (6.9%)	–	51 (6.7%)
Disease activity				
PGA (NRS 0–10), mean (SD)	3.8 (2.6)	1.0 (1.5)	0.8 (1.4)	0.8 (1.3)
cJADAS-10 (0–30), mean (SD)	9.8 (6.2)	3.4 (4.0)	2.8 (3.4)	2.7 (3.6)
Inactive disease, *n* (%)	56 (6.0%)	288 (39.8%)	309 (46.8%)	297 (52.0%)
Minimal disease activity, *n* (%)	24 (2.6%)	89 (12.3%)	85 (12.9%)	64 (11.2%)
Moderate disease activity, *n* (%)	159 (17.0%)	218 (30.2%)	175 (26.5%)	132 (23.1%)
High disease activity, *n* (%)	698 (74.5%)	128 (17.7%)	92 (13.9%)	78 (13.7%)
Parent’s reported outcomes				
Functional status (CHAQ; range 0–3), mean (SD)	0.6 (0.7)	0.3 (0.5)	0.2 (0.5)	0.2 (0.5)
FaBel (total scale; range 0–4), mean (SD)^a^	1.7 (0.4)	–	–	–
Treatment				
NSAIDs, *n* (%)	800 (84.0%)	289 (37.8%)	191 (26.1%)	150 (22.4%)
Systemic glucocorticoids, *n* (%)	230 (24.1%)	83 (10.9%)	49 (6.7%)	32 (4.8%)
DMARDs, *n* (%)	394 (41.3%)	493 (64.5%)	427 (58.3%)	390 (58.2%)
csDMARDs, *n* (%)	387 (40.6%)	454 (59.4%)	350 (47.8%)	313 (46.7%)
MTX, *n* (%)	364 (38.2%)	422 (55.2%)	324 (44.2%)	285 (42.5%)
Sulfasalazine, *n* (%)	19 (2%)	22 (2.9%)	12 (1.6%)	11 (1.6%)
bDMARDs, *n* (%)	38 (4.0%)	156 (20.4%)	179 (24.4%)	163 (24.3%)
Etanercept, *n* (%)	21 (2.2%)	86 (11.3%)	105 (14.3%)	86 (12.8%)
Adalimumab, *n* (%)	7 (0.7%)	35 (4.6%)	37 (5.1%)	46 (6.9%)

bDMARD, biologic DMARD; CHAQ, Childhood Health Assessment Questionnaire; cJADAS-10, clinical Juvenile Arthritis Disease Activity Score; csDMARD, conventional synthetic DMARD; DMARD, disease-modifying antirheumatic drug; FaBel, Family Burden Questionnaire; JIA, juvenile idiopathic arthritis; MTX, methotrexate; NRS, numeric rating scale; NSAID, nonsteroidal anti-inflammatory drug; PGA, physician’s global assessment of disease activity; RF, rheumatoid factor; SD, standard deviation

^a^Reported 3 months after baseline

Linear mixed models were applied to compare the primary outcome (PedsQL scores) between the groups. We applied this model to yield unbiased estimates in the presence of missing data. A characteristic of these models is that observations with partly missing data are not discarded from the analysis and will contribute to the estimate of the overall mean response.

To set thresholds for the suboptimal HRQoL we used the data from our German weighted peer sample. To facilitate a simple interpretation, the threshold in the PedsQL was defined as the point which was exceeded by 90% of the peers. Ninety percent of the peers had a PedsQL total score of 79.3 or above, a PedsQL physical health score of 84.4 or above, and a psychosocial health score of 73.3 or above. We used these values as thresholds to differentiate between a favorable or high quality of life, and suboptimal HRQoL. Univariable and multiple backward logistic regressions were used to identify the predictors of suboptimal HRQoL at 3-year FU. Age was included in the multiple analyses to adjust for the dropout of older children during the study course.

Linear regression analyses were applied to investigate associations between HRQoL and sociodemographic variables, parent-reported outcomes, and disease-specific clinical outcomes at the same point in time.

Patients with active disease at enrollment (cJADAS-10 > 1) were stratified into groups with minimal, moderate, high, and very high improvement. The quartiles and median changes in cJADAS-10 between enrollment and the 36-month visit were used to calculate the corresponding cut-off points (3, 6.5, and 11.5). Linear mixed models were used to investigate the PedsQL outcome in the JIA subgroups (JIA categories, groups of cJADAS-10 improvement). Means and frequencies of other secondary outcomes (e.g., disease activity, pain, and physical disability) were provided as observed.

## Results

### Baseline characteristics and comparisons between JIA patients and healthy peers

**Fig. 1:**
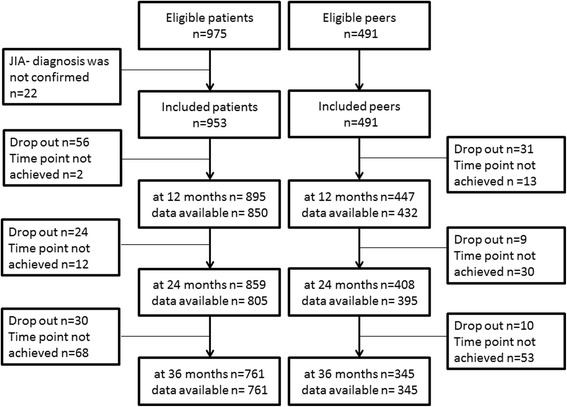
Disposition of ICON participants included in the present analysis. JIA, juvenile idiopathic arthritis

Data from 953 patients with newly diagnosed JIA and 491 healthy peers were included in the analysis. Dropout rates over the observation period were low and ranged between 2% and 6% per year (Fig. 1). Baseline characteristics of both groups differed significantly regarding health-related parameters, as well as for age, sex, and SES (Table 1). An adjustment was made (see the Statistical analysis section above) for this reason. Comparisons between both groups are based on the resulting weighted samples. Compared with the healthy peers, the HRQoL in JIA patients was impaired in both physical and psychosocial health (Table 1). Significantly higher SDQ total scores were found in JIA patients. JIA patients with borderline or conspicuous psychosocial problems at enrollment (SDQ total score ≥ 14) had a lower HRQoL (mean total score = 59, standard deviation (SD) 16). In multivariate linear regression analyses, the PedsQL total score was significantly associated with the SDQ total score and the parent’s global score in both groups, and with pain in patients with JIA (all *p* < 0.001).

### Characteristics of JIA patients, disease activity, and treatment from enrollment to 36 months

The mean age at JIA diagnosis was 7.7 years (SD 4.8). The median time from JIA diagnosis to enrollment was 2 months (interquartile range (IQR) 0–4). A history of uveitis or psoriasis was present in 5.9% and 3.5% of patients at enrollment, respectively. Nearly half of the patients had oligoarthritis (OA), and one-quarter had RF-negative polyarthritis (Table 2). At enrollment, categorization into persistent and extended OA was possible only in a minority of patients. Among these, 25 were diagnosed as having extended OA and, during follow-up, another 78 developed an extended disease course. Furthermore, in 16 children the JIA category was changed mainly due to the onset of psoriasis, enthesitis, sacroiliitis, or dactylitis, or new information on the onset of psoriasis in first-degree relatives.

Prior to, and at enrollment, almost all patients had received nonsteroidal anti-inflammatory drugs (NSAIDs), and 48% were treated with intra-articular glucocorticoids. Prior to their inclusion in the ICON study, treatment with conventional synthetic disease-modifying antirheumatic drugs (csDMARDs) was initiated in 44.2% of patients, whereas 55.5% were DMARD naive. This treatment pattern changed within the first 6 to 12 months after enrollment. About one-quarter were treated with tumor necrosis factor (TNF) inhibitors or other biologic DMARDs (bDMARDs) at the 2-year and 3-year FU. Intra-articular glucocorticoids were used in 11% of the patients within 6 months of the 2- and 3-year FU. Treatment was accompanied by a significant improvement in the HRQoL (PedsQL scores) of patients, disease activity, and functional ability (CHAQ). The mean cJADAS-10 reduced significantly within the first 6 months of standard care from 9.8 to 4.2 (SD 4.2). Half of the JIA patients were in a clinically inactive disease state at their 3-year FU (Table 2).

### Course of HRQoL over 36 months, comparing children with JIA with healthy peers

**Fig. 2:**
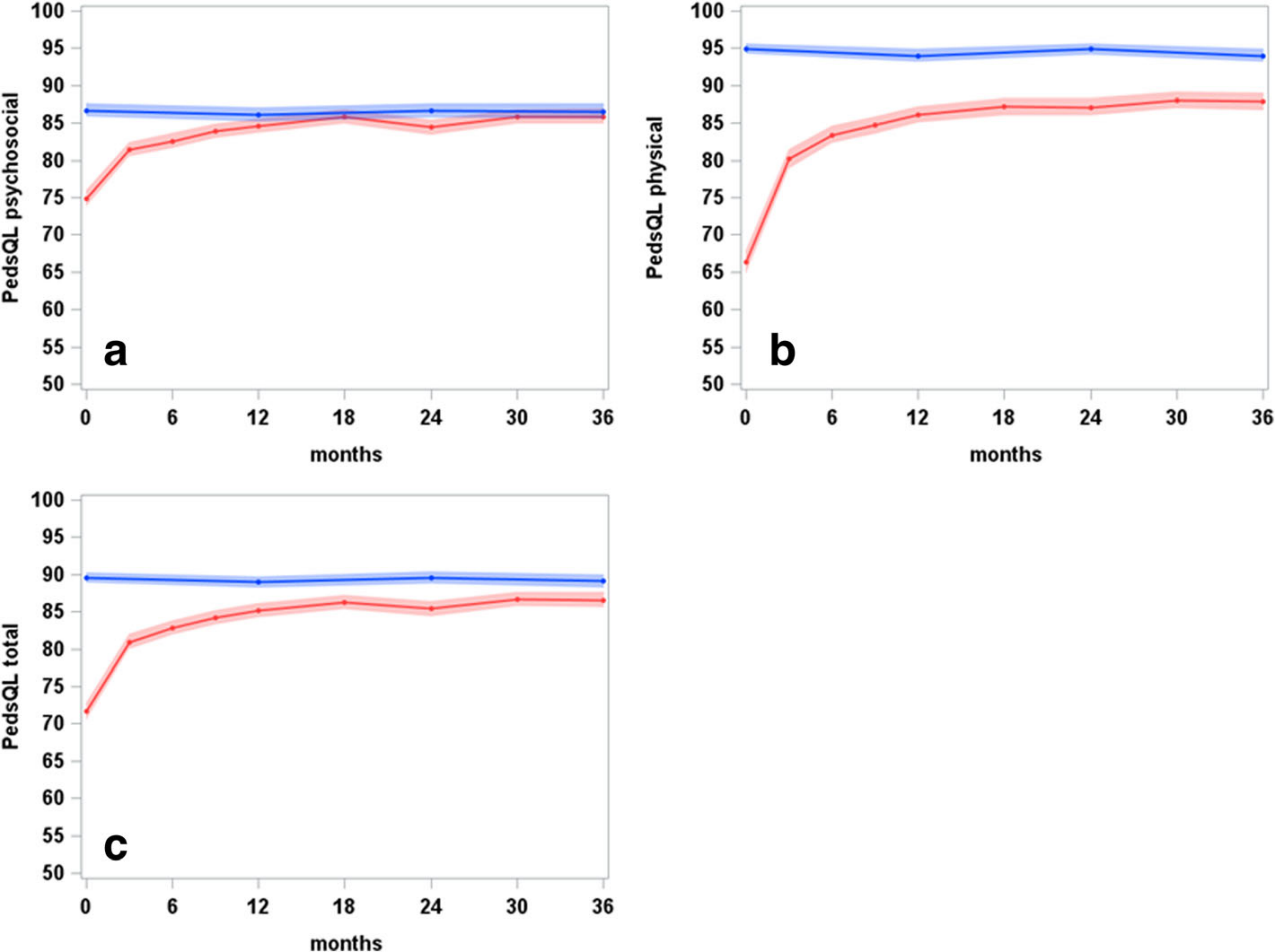
Least square means of (**a**) Pediatric Quality of Life Inventory (PedsQL) psychosocial scores, **b** PedsQL physical scores, and (**c**) PedsQL total scores, and corresponding 95% confidence bands, of JIA patients (red) and healthy peers (blue) over 3 years of observation in ICON (results of linear mixed model analyses)

Over the first 3 years of rheumatologic care, the HRQoL improvement in patients was statistically significant. On average, improvements of 11.2 (95% confidence interval (CI) 10.1–12.3), 21.7 (20.2–23.2), and 15.0 (13.9–16.1) units were observed in the psychosocial, physical health, and PedsQL total scores, respectively. There was a sharp increase in the PedsQL scores of the JIA patients within the first 6 months. The mean psychosocial health scores of patients with JIA were equivalent to those of their healthy peers (Fig. 2a). Mean significant differences between JIA patients and healthy peers for physical health and PedsQL total scores of 5.8 (95% CI 4.1–7.6) and 2.4 (0.9–4.0) remained (Fig. 2b, c).

Decreased disease activity was accompanied by an improvement in HRQoL. JIA patients with an inactive disease status at 3-year FU had a higher mean PedsQL total score of 92.8 (SD 8.8) than those with moderate (80.4 (16.0)) or high disease activity (73.6 (17.4)). A similar result was found by comparing the change in cJADAS with the change in PedsQL, which both depend on the baseline level. In patients with minimal improvement (see Statistical analysis section) in cJADAS-10 (32.7%), the PedsQL total score increased from 79.1 (95% CI 76.7–81.5) at enrollment to 83.1 (81.0–85.1) at 3-year FU; in those with moderate improvement (19.9%), it increased from 75.5 (72.5–78.5) to 87.7 (85.2–90.2); in patients with high improvement (24.2%), it increased from 68.6 (65.9–71.4) to 88.4 (86.1–90.8); and in those with very high improvement (23.1%), it increased from 59.8 (56.9–62.8) to 89.3 (86.9–91.8).

### Changes in HRQoL in children with different JIA categories

**Fig. 3:**
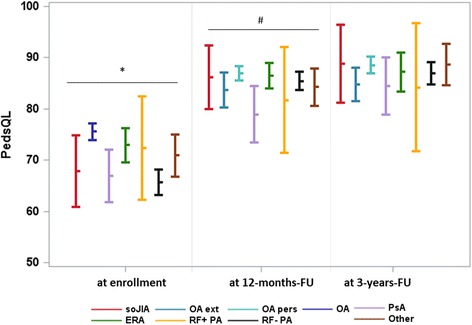
HRQoL of patients with JIA differentiated by JIA category at baseline, at 12-month follow-up (FU), and at 3-year FU. Values are shown as least square means with corresponding 95% confidence intervals. Patients were assigned to the JIA category they refer to at the corresponding point in time. ERA, enthesitis-related arthritis; OA, oligoarthritis; OA ext, extended oligoarthritis; OA pers, persistent oligoarthritis; PedsQL, Pediatric Quality of Life Inventory; PsA, psoriatic arthritis; RF+ PA, rheumatoid factor-positive polyarthritis; RF- PA, rheumatoid factor-negative polyarthritis; soJIA, systemic-onset juvenile idiopathic arthritis. *p < 0.001, ^#^*p* < 0.001

At enrollment, the PedsQL total score differed significantly between JIA categories (*p* < 0.001). In particular, patients with RF-negative polyarthritis, psoriatic arthritis, and systemic arthritis reported a low HRQoL at baseline. An improvement in HRQoL was observed in all JIA categories, but to varying degrees (Fig. 3). The change in HRQoL was more distinct in patients with RF-negative polyarthritis and systemic JIA and was less pronounced in patients with psoriatic arthritis. At 12-month FU, differences between the JIA categories were still significant (*p* = 0.03), with ranges in PedsQL total score from 79.2 to 87.1. In particular, patients with psoriatic arthritis differed from others due to their low HRQoL. There was no statistically significant (*p* = 0.44) difference in HRQoL at the 3-year FU.

### Predictors of suboptimal HRQoL at the 3-year FU

Lower HRQoL at the 36-month FU was strongly associated with higher pain, increased functional disability, and worse parent global score at baseline (all *p* values < 0.01, *R*^2^ = 0.54) in a multivariable linear stepwise regression.

Using the PedsQL data of the peers to define a favorable HRQoL (score ≥ 79.3), we found that 76% of patients with JIA attained this HRQoL, with a mean PedsQL total value of 93.6 (SD 6.3). Those who did not achieve this threshold had a mean total score of 66.1 (11.4). With regard to physical and psychosocial health, 70% versus 82% of patients reached a favorable HRQoL (scores ≥ 84.4 and ≥ 73.3, respectively), with mean PedsQL physical health and psychosocial health values of 96.9 (4.3) and 91.9 (8.1). Mean levels of 68.2 (15.5) and 61.0 (10.4) were observed in children with suboptimal physical and psychosocial health, respectively. Associations between baseline parameters and the likelihood of a suboptimal HRQoL at the 36-month FU are shown in Table 3. The FaBel total score had a consistently large effect on poor HRQoL in multivariable regression analyses. In addition, subjectively perceived pain and the parent global score were significantly associated. Overall, a higher burden on the family, increased pain, and a worse parent global score at enrollment increased the likelihood of suboptimal HRQoL after 3 years of rheumatologic care.

**Table 3:** Predictors at enrollment of a suboptimal HRQoL at the 36-month follow-up

Predictors	PedsQL score of suboptimal HRQoL at the 36-month follow up			
	Total < 79.3		Physical health < 84.4	Psychosocial health < 73.3
	Univariate	Multiple	Multiple	Multiple
Age^a^	1.01 (0.97–1.05)	1.02 (0.97–1.07)	1.06 (1.02–1.11)**	1.04 (0.99–1.09)
Sex^b^	1.01 (0.70–1.45)		0.57 (0.38–0.85)**	
SES score	0.97 (0.92–1.03)			
Time between symptom onset and diagnosis	1.01 (1.00–1.03)			
Uveitis	0.86 (0.41–1.66)			
JIA categories				
Persistent oligoarthritis	Referent			
Extended oligoarthritis	1.58 (0.91–2.71)			
RF-negative polyarthritis	1.10 (0.69–1.73)			
RF-positive polyarthritis	2.24 (0.63–7.22)			
Psoriatic arthritis	1.53 (0.66–3.36)			
Enthesitis-related arthritis	0.95 (0.48–1.80)			
Systemic-onset JIA	1.22 (0.42–3.12)			
Undifferentiated arthritis	0.80 (0.34–1.71)			
Disease activity				
cJADAS-10	1.03 (1.00–1.06)*			
PGA	1.02 (0.96–1.09)			
Parent’s global	1.19 (1.10–1.29)***	1.15 (1.05–1.26)**		1.14 (1.03–1.26)**
Active joint count	1.00 (0.97–1.02)			
Pain				
Numeric rating scale	1.14 (1.07–1.22)***		1.21 (1.13–1.30)***	
Functional ability				
CHAQ	1.74 (1.36–2.23)***			
Psychosocial burden of families				
FaBel total	4.82 (2.96–7.98)***	3.88 (2.27– 6.72)***	2.97 (1.78–5.02)***	3.63 (2.04–6.53)***

*p < 0.05, **p < 0.01, ***p < 0.0001

CHAQ, Childhood Health Assessment Questionnaire; cJADAS-10, clinical Juvenile Arthritis Disease Activity Score; FaBel, Family Burden Questionnaire; HRQoL, health-related quality of life; JIA, juvenile idiopathic arthritis; PedsQL, Pediatric Quality of Life Inventory; PGA, physician’s global assessment of disease activity; RF, rheumatoid factor; SES, socioeconomic status

^a^ Admitted to multivariate analyses to adjust for missing data

^b^ Female referent

## Discussion

Our results confirm earlier findings using the ICON cohort of a noticeable reduction in disease activity in early JIA which can currently be achieved within the first 6 months of routine rheumatologic care [26]. Half of the JIA patients achieved an inactive disease status after 3 years of treatment if the cJADAS-10, which also includes the parent’s perspective, is used for assessment. A greater decrease in cJADAS-10 was associated with a greater increase in HRQoL. Parent-reported functional disability, pain, and parent global scores were significantly associated with HRQoL. The level of psychosocial health of JIA patients was comparable with that of healthy peers after 3 years of rheumatologic care, whereas a minor but significant difference remained in physical health. Different JIA categories showed unique HRQoL trajectories but, at 3-year FU, the PedsQL total scores had reached a comparable level in all categories. Three-quarters of patients with JIA reached a favorable HRQoL. Predictors of a suboptimal HRQoL (PedsQL total score < 79.3) were the parent global and pain scores at enrollment, as well as the burden borne by the family as a result of their child’s illness.

Different methods are applied to measure disease activity in JIA. We used the cJADAS-10 because of its multidimensional concept. Our findings are consistent with those of others who also observed a clear decrease in disease activity and functional limitations following treatment for recent-onset JIA [20, 39, 40].

Many studies found a lower HRQoL in patients with JIA compared with healthy controls [4, 8–15, 41]. However, their results cannot simply be transferred to recent-onset JIA patients treated in pediatric rheumatologic care since patients were often sampled in the pre-biologic era of the 1980s or 1990s [8, 11, 41] or had a disease duration of more than 10 years [4, 8, 11, 41]. It should also be considered that most of these studies were cross-sectional in nature [8, 12–15, 41] and therefore did not permit conclusions about the HRQoL in JIA patients during the course of their disease.

After 36 months of rheumatologic care, we observed a clear approximation of the patients’ PedsQL values to those of healthy peers. Regarding psychosocial health, our findings are in accordance with prior studies that found that psychosocial health was similar in patients with JIA and healthy peers [4, 10, 41, 42]. It is in line with other studies on the HRQoL of patients with JIA [4, 15] that physical health is more affected than psychosocial health, which was also shown in our analysis. Findings from the literature suggest that parents of children with health problems tend to underestimate their child’s HRQoL, whereas parents from nonclinical samples tend to report a higher HRQoL than the children themselves [43]. Considering these findings, we suppose that the convergence of PedsQL values of JIA patients to levels of age-, sex-, and SES-comparable peers is more likely to be under- rather than overestimated in our cohort.

Until now, longitudinal studies on the HRQoL of children with JIA are rare [4, 16, 19]. Oen et al. [19] investigated the HRQoL of 1249 newly diagnosed patients with JIA using the Juvenile Arthritis QoL Questionnaire (JAQQ; range 1–7). They described an improvement in median JAQQ score from 2.7 at the first visit to 1.9 at 7 months and 1.5 at 37 months after diagnosis. Similar to our data, an improvement in HRQoL was most marked within the first 6 months. We identified mean HRQoL changes that were two- to three-times higher than the corresponding minimal clinically important different PedsQL scores calculated by Varni et al. [33]. This underlines the clear clinically relevant improvement in HRQoL that was already achieved during the first months of treatment and whose level could be maintained over time.

In the ICON cohort, an improvement in HRQoL to the level measured in healthy peers was observed in each JIA category. At enrollment, each disease category differed significantly in their PedsQL total score, with particularly low scores in those patients with systemic JIA and RF-negative polyarthritis. At the onset of disease, systemic JIA is characterized by daily spiking fevers and is accompanied by a variety of other systemic signs [27] associated with a high disease burden and a decreased quality of life [8]. Patients with RF-negative polyarthritis may have low PedsQL scores at disease onset because of the restricted mobility caused by the inflammation in more than four joints. After 3 years, there were no relevant differences in HRQoL between the various JIA categories.

Our results are consistent with those reported by Oen et al. [19], who observed equivocal quality of life scores in the different JIA categories within 50 months of diagnosis. In our findings, this convergence was reached 12 months earlier. The almost equal quality of life of the different JIA categories was not found 10 years ago [8, 12, 14], which may be due to the improved effects of new treatment options. In the ICON cohort, patients with JIA were intensively treated (see [26]) according to current treatment recommendations [44–46]. Within the first year of observation, patients with systemic arthritis who received an interleukin (IL)-1 or IL-6 inhibitor most commonly achieved an inactive disease status [26] and had the highest mean PedsQL total score at the 1-year FU. In contrast, patients with psoriatic arthritis reached an inactive disease state less frequently [26] and had the lowest HRQoL scores at FU. Earlier findings [4, 41, 47] of a better HRQoL in JIA patients with inactive disease were confirmed in our study. The association between HRQoL and the clinical parameters of JIA have already been investigated in several studies [4, 9, 11, 12, 22, 48]. Significant associations between HRQoL and disease activity, physical disability, and pain have been described [4, 9, 22]. The impact of disability and pain was found to be stronger than that of disease activity in general, which is in line with our findings. Patient-reported (or, as in our study, parent-reported) outcomes were particularly associated with the patient’s HRQoL, while clinical parameters such as the physician’s global score or the number of actively involved joints played a minor role.

The proportion of JIA patients with a suboptimal HRQoL was investigated in other studies [9, 25, 49] which used a similar PedsQL cut-off to our work (< 78.6 versus < 79.3) to differentiate between optimal and suboptimal HRQOL. Lundberg et al. [49] and Haverman et al. [9] observed a suboptimal HRQoL in nearly half of their patients with JIA after a median of 2–5 and 3.6 years of disease duration, respectively. In ICON, the outcome was more favorable. Only a quarter of the patients with JIA had a suboptimal HRQoL after 3 years of rheumatologic care. We identified pain and the parent’s global assessment at enrollment as predictors of a suboptimal HRQoL status at FU. This is similar to the findings of others [4, 21, 50]. We further found that the burden caused by the disease on the child’s family is another important predictor of a suboptimal quality of life. This is in accordance with other findings [24, 51] that described an association between HRQoL and the child’s perception of social support and parental distress.

The strength of this study is the large sample size that accounts for differences in sociodemographic parameters such as age, sex, and social status between JIA patients and healthy peers. Only proxy-reported HRQoL was used to make conclusions for the whole cohort over the entire age range, rather than only in older patients and peers by self-reported HRQoL. It should be considered that proxy-reported HRQoL estimates may be biased compared with self-reports. However, the agreement between data reported by children and their parents is often high, especially in the case of sick children [47, 52]. In cases of discordance, the parents of sick patients described on average a lower HRQoL than the children themselves [43], resulting in an underestimation in HRQoL. There is another limitation that should not go unmentioned. We did not investigate the impact of individual treatment modalities on quality of life outcomes, as the ICON is an observational cohort study and treatment decisions prior to or after enrollment were confounded by their clinical indication.

## Conclusion

With current treatment strategies, it is possible to achieve a favorable quality of life in most patients with JIA. After 3 years of pediatric rheumatology care, JIA patients have almost the same quality of life as healthy peers. This is a crucial finding for the counseling of patients and their parents. Among characteristics assessed at enrollment, family burden, pain, and functional impairment were more important than disease activity as predictors of a suboptimal quality of life over the course of this study. It is therefore important to look for these risk factors in clinical practice to be able to set the course at an early stage of the disease with targeted support measures.

## Abbreviations

ANA: Antinuclear antibodies
bDMARD: Biologic disease-modifying antirheumatic drug
CHAQ: Childhood Health Assessment Questionnaire
CI: Confidence interval
cJADAS: Clinical Juvenile Arthritis Disease Activity Score
CRP: C-reactive protein
csDMARD: Conventional synthetic disease-modifying antirheumatic drug
DMARD: Disease-modifying antirheumatic drug
ESR: Erythrocyte sedimentation rate
FaBel: Family Burden Questionnaire
FU: Follow-up
HLA-B27: Human leukocyte antigen-B27
HRQoL: Health-related quality of life
ICON: Inception cohort of patients with newly diagnosed juvenile idiopathic arthritis
IL: Interleukin
ILAR: International League of Associations for Rheumatology
JAQQ: Juvenile Arthritis Quality of Life Questionnaire
JIA: Juvenile idiopathic arthritis
NRS: Numeric rating scale
NSAID: Nonsteroidal anti-inflammatory drug
OA: Oligoarthritis
PedsQL: Pediatric Quality of Life Inventory
PGA: Physician’s global assessment of disease activity
RF: Rheumatoid factor
SD: Standard deviation
SDQ: Strength and Difficulties Questionnaire
SES: Socioeconomic status

## References

[1] Prakken B, Albani S, Martini A (2011). Juvenile idiopathic arthritis. Lancet.

[2] Magni-Manzoni S, Pistorio A, Labo E, Viola S, Garcia-Munitis P, Panigada S, Visconti C, Buoncompagni A, Martini A, Ravelli A (2008). A longitudinal analysis of physical functional disability over the course of juvenile idiopathic arthritis. Ann Rheum Dis.

[3] Packham JC, Hall MA (2002). Long-term follow-up of 246 adults with juvenile idiopathic arthritis: functional outcome. Rheumatology (Oxford).

[4] Tollisen A, Selvaag AM, Aulie HA, Lilleby V, Aasland A, Lerdal A, Flato B. Physical functioning, pain and health-related quality of life in adults with juvenile idiopathic arthritis: a longitudinal 30-year follow-up study. Arthritis Care Res (Hoboken). 2017;. 10.1002/acr.23327. [Epub ahead of print] 10.1002/acr.23327 28732134

[5] Bullinger M, Hasford J (1991). Evaluating quality-of-life measures for clinical trials in Germany. Control Clin Trials.

[6] The World Health Organization Quality of Life assessment (WHOQOL): position paper from the World Health Organization. Soc Sci Med. 1995;41(10):1403–9. 10.1016/0277-9536(95)00112-k 8560308

[7] Saxena S, Orley J, Group W (1997). Quality of life assessment: The World Health Organization perspective. Eur Psychiatry.

[8] Foster HE, Marshall N, Myers A, Dunkley P, Griffiths ID (2003). Outcome in adults with juvenile idiopathic arthritis: a quality of life study. Arthritis Rheum.

[9] Haverman L, Grootenhuis MA, van den Berg JM, van Veenendaal M, Dolman KM, Swart JF, Kuijpers TW, van Rossum MA (2012). Predictors of health-related quality of life in children and adolescents with juvenile idiopathic arthritis: results from a web-based survey. Arthritis Care Res (Hoboken).

[10] Manczak M, Rutkowska-Sak L, Raciborski F (2016). Health-related quality of life in children with juvenile idiopathic arthritis—child's and parent's point of view. Reumatologia.

[11] Bertilsson L, Andersson-Gare B, Fasth A, Petersson IF, Forsblad-D'elia H (2013). Disease course, outcome, and predictors of outcome in a population-based juvenile chronic arthritis cohort followed for 17 years. J Rheumatol.

[12] Oliveira S, Ravelli A, Pistorio A, Castell E, Malattia C, Prieur AM, Saad-Magalhaes C, Murray KJ, Bae SC, Joos R (2007). Proxy-reported health-related quality of life of patients with juvenile idiopathic arthritis: the Pediatric Rheumatology International Trials Organization multinational quality of life cohort study. Arthritis Rheum.

[13] Barth S, Haas JP, Schlichtiger J, Molz J, Bisdorff B, Michels H, Hugle B, Radon K (2016). Long-term health-related quality of life in German patients with juvenile idiopathic arthritis in comparison to German general population. PLoS One.

[14] Gutierrez-Suarez R, Pistorio A, Cespedes Cruz A, Norambuena X, Flato B, Rumba I, Harjacek M, Nielsen S, Susic G, Mihaylova D (2007). Health-related quality of life of patients with juvenile idiopathic arthritis coming from 3 different geographic areas. The PRINTO multinational quality of life cohort study. Rheumatology (Oxford).

[15] Solari N, Viola S, Pistorio A, Magni-Manzoni S, Vitale R, Ruperto N, Ullmann N, Filocamo G, Martini A, Ravelli A (2008). Assessing current outcomes of juvenile idiopathic arthritis: a cross-sectional study in a tertiary center sample. Arthritis Rheum.

[16] Anink J, Prince FH, Dijkstra M, Otten MH, Twilt M, ten Cate R, Gorter SL, Koopman-Keemink Y, van Rossum MA, Hoppenreijs EP (2015). Long-term quality of life and functional outcome of patients with juvenile idiopathic arthritis in the biologic era: a longitudinal follow-up study in the Dutch Arthritis and Biologicals in Children Register. Rheumatology (Oxford).

[17] Prince FH, Geerdink LM, Borsboom GJ, Twilt M, van Rossum MA, Hoppenreijs EP, Cate RT, Koopman-Keemink Y, van Santen-Hoeufft M, Raat H (2010). Major improvements in health-related quality of life during the use of etanercept in patients with previously refractory juvenile idiopathic arthritis. Ann Rheum Dis.

[18] Ruperto N, Lovell DJ, Li T, Sztajnbok F, Goldenstein-Schainberg C, Scheinberg M, Penades IC, Fischbach M, Alcala JO, Hashkes PJ (2010). Abatacept improves health-related quality of life, pain, sleep quality, and daily participation in subjects with juvenile idiopathic arthritis. Arthritis Care Res (Hoboken).

[19] Oen K, Guzman J, Dufault B, Tucker LB, Shiff NJ, Watanabe Duffy K, Lee JJ, Feldman BM, Berard RA, Dancey P, et al. Health-related quality of life in an inception cohort of children with juvenile idiopathic arthritis: a longitudinal analysis. Arthritis Care Res (Hoboken). 2018;70(1):134-44. 10.1002/acr.23236. Epub 2017 Dec 6. 10.1002/acr.23236 28320056

[20] Nordal E, Zak M, Aalto K, Berntson L, Fasth A, Herlin T, Lahdenne P, Nielsen S, Straume B, Rygg M (2011). Ongoing disease activity and changing categories in a long-term nordic cohort study of juvenile idiopathic arthritis. Arthritis Rheum.

[21] Klotsche J, Minden K, Thon A, Ganser G, Urban A, Horneff G (2014). Improvement in health-related quality of life for children with juvenile idiopathic arthritis after start of treatment with etanercept. Arthritis Care Res (Hoboken).

[22] Shaw KL, Southwood TR, Duffy CM, McDonagh JE (2006). Health-related quality of life in adolescents with juvenile idiopathic arthritis. Arthritis Rheum.

[23] van Dijkhuizen EH, Wulffraat NM (2015). Early predictors of prognosis in juvenile idiopathic arthritis: a systematic literature review. Ann Rheum Dis.

[24] Seid M, Huang B, Niehaus S, Brunner HI, Lovell DJ (2014). Determinants of health-related quality of life in children newly diagnosed with juvenile idiopathic arthritis. Arthritis Care Res (Hoboken).

[25] Seid M, Opipari L, Huang B, Brunner HI, Lovell DJ (2009). Disease control and health-related quality of life in juvenile idiopathic arthritis. Arthritis Rheum.

[26] Sengler C, Klotsche J, Niewerth M, Liedmann I, Foll D, Heiligenhaus A, Ganser G, Horneff G, Haas JP, Minden K (2015). The majority of newly diagnosed patients with juvenile idiopathic arthritis reach an inactive disease state within the first year of specialised care: data from a German inception cohort. RMD Open.

[27] Petty RE, Southwood TR, Manners P, Baum J, Glass DN, Goldenberg J, He X, Maldonado-Cocco J, Orozco-Alcala J, Prieur AM (2004). International League of Associations for Rheumatology classification of juvenile idiopathic arthritis: second revision, Edmonton, 2001. J Rheumatol.

[28] McErlane F, Beresford MW, Baildam EM, Chieng SE, Davidson JE, Foster HE, Gardner-Medwin J, Lunt M, Wedderburn LR, Thomson W (2013). Validity of a three-variable juvenile arthritis disease activity score in children with new-onset juvenile idiopathic arthritis. Ann Rheum Dis.

[29] Consolaro A, Bracciolini G, Ruperto N, Pistorio A, Magni-Manzoni S, Malattia C, Pederzoli S, Davi S, Martini A, Ravelli A (2012). Remission, minimal disease activity, and acceptable symptom state in juvenile idiopathic arthritis: defining criteria based on the juvenile arthritis disease activity score. Arthritis Rheum.

[30] Consolaro A, Ravelli A (2016). Defining criteria for disease activity states in juvenile idiopathic arthritis. Rheumatology (Oxford).

[31] Singh G, Athreya BH, Fries JF, Goldsmith DP (1994). Measurement of health status in children with juvenile rheumatoid arthritis. Arthritis Rheum.

[32] Varni JW, Seid M, Smith Knight T, Burwinkle T, Brown J, Szer IS (2002). The PedsQL in pediatric rheumatology: reliability, validity, and responsiveness of the Pediatric Quality of Life Inventory Generic Core Scales and Rheumatology Module. Arthritis Rheum.

[33] Varni JW, Burwinkle TM, Seid M, Skarr D (2003). The PedsQL 4.0 as a pediatric population health measure: feasibility, reliability, and validity. Ambul Pediatr.

[34] Goodman R (1997). The Strengths and Difficulties Questionnaire: a research note. J Child Psychol Psychiatry.

[35] Stone LL, Janssens JM, Vermulst AA, Van Der Maten M, Engels RC, Otten R (2015). The Strengths and Difficulties Questionnaire: psychometric properties of the parent and teacher version in children aged 4-7. BMC Psychol.

[36] Stein RE, Riessman CK (1980). The development of an impact-on-family scale: preliminary findings. Med Care.

[37] Ravens-Sieberer U, Morfeld M, Stein RE, Jessop DJ, Bullinger M, Thyen U (2001). The testing and validation of the German version of the impact on family scale in families with children with disabilities. Psychother Psychosom Med Psychol.

[38] Lampert T, Muters S, Stolzenberg H, Kroll LE, Ki GGSSG (2014). Measurement of socioeconomic status in the KiGGS study: first follow-up (KiGGS Wave 1). Bundesgesundheitsblatt Gesundheitsforschung Gesundheitsschutz.

[39] McErlane F, Foster HE, Carrasco R, Baildam EM, Chieng SE, Davidson JE, Ioannou Y, Wedderburn LR, Thomson W, Hyrich KL (2016). Trends in paediatric rheumatology referral times and disease activity indices over a ten-year period among children and young people with juvenile idiopathic arthritis: results from the childhood arthritis prospective Study. Rheumatology (Oxford).

[40] Oen K, Duffy CM, Tse SM, Ramsey S, Ellsworth J, Chedeville G, Chetaille AL, Saint-Cyr C, Cabral DA, Spiegel LR (2010). Early outcomes and improvement of patients with juvenile idiopathic arthritis enrolled in a Canadian multicenter inception cohort. Arthritis Care Res (Hoboken).

[41] Flato B, Lien G, Smerdel A, Vinje O, Dale K, Johnston V, Sorskaar D, Moum T, Ploski R, Forre O (2003). Prognostic factors in juvenile rheumatoid arthritis: a case-control study revealing early predictors and outcome after 14.9 years. J Rheumatol.

[42] Noll RB, Kozlowski K, Gerhardt C, Vannatta K, Taylor J, Passo M (2000). Social, emotional, and behavioral functioning of children with juvenile rheumatoid arthritis. Arthritis Rheum.

[43] Upton P, Lawford J, Eiser C (2008). Parent-child agreement across child health-related quality of life instruments: a review of the literature. Qual Life Res.

[44] Ringold S, Weiss PF, Beukelman T, DeWitt EM, Ilowite NT, Kimura Y, Laxer RM, Lovell DJ, Nigrovic PA, Robinson AB (2013). 2013 update of the 2011 American College of Rheumatology recommendations for the treatment of juvenile idiopathic arthritis: recommendations for the medical therapy of children with systemic juvenile idiopathic arthritis and tuberculosis screening among children receiving biologic medications. Arthritis Rheum.

[45] Dueckers G, Guellac N, Arbogast M, Dannecker G, Foeldvari I, Frosch M, Ganser G, Heiligenhaus A, Horneff G, Illhardt A (2012). Evidence and consensus based GKJR guidelines for the treatment of juvenile idiopathic arthritis. Clin Immunol.

[46] Beukelman T (2014). Treatment advances in systemic juvenile idiopathic arthritis. F1000Prime Rep.

[47] Ringold S, Wallace CA, Rivara FP (2009). Health-related quality of life, physical function, fatigue, and disease activity in children with established polyarticular juvenile idiopathic arthritis. J Rheumatol.

[48] Mulligan K, Kassoumeri L, Etheridge A, Moncrieffe H, Wedderburn LR, Newman S (2013). Mothers’ reports of the difficulties that their children experience in taking methotrexate for juvenile idiopathic arthritis and how these impact on quality of life. Pediatr Rheumatol Online J.

[49] Lundberg V, Lindh V, Eriksson C, Petersen S, Eurenius E (2012). Health-related quality of life in girls and boys with juvenile idiopathic arthritis: self- and parental reports in a cross-sectional study. Pediatr Rheumatol Online J.

[50] Taxter AJ, Wileyto EP, Behrens EM, Weiss PF (2015). Patient-reported outcomes across categories of juvenile idiopathic arthritis. J Rheumatol.

[51] Aasland A, Flato B, Vandvik IH (1997). Psychosocial outcome in juvenile chronic arthritis: a nine-year follow-up. Clin Exp Rheumatol.

[52] April KT, Feldman DE, Platt RW, Duffy CM (2006). Comparison between children with juvenile idiopathic arthritis (JIA) and their parents concerning perceived quality of life. Qual Life Res.

